# Diversity of Cacao Trees in Waslala, Nicaragua: Associations between Genotype Spectra, Product Quality and Yield Potential

**DOI:** 10.1371/journal.pone.0054079

**Published:** 2013-01-18

**Authors:** Bodo Trognitz, Emile Cros, Sophie Assemat, Fabrice Davrieux, Nelly Forestier-Chiron, Eusebio Ayestas, Aldo Kuant, Xavier Scheldeman, Michael Hermann

**Affiliations:** 1 Dept. Health and Environment, AIT Austrian Institute of Technology, Tulln, Austria; 2 Plant Growth and Phenotyping Facility, CSF Campus Science Support Facilities, Vienna, Austria; 3 Dept. Persyst Performances des Systèmes de Production et de Transformation Tropicaux, CIRAD Centre de coopération internationale en recherche agronomique pour le développement, Montpellier, France; 4 Facultad de Agronomía, Universidad Nacional Agraria, Managua, Nicaragua; 5 Pro Mundo Humano, Waslala, Nicaragua; 6 Regional Office for the Americas, Bioversity International, Cali, Colombia; 7 Crops for the Future, Serdang, Malaysia; CNR, Italy

## Abstract

The sensory quality and the contents of quality-determining chemical compounds in unfermented and fermented cocoa from 100 cacao trees (individual genotypes) representing groups of nine genotype spectra (GG), grown at smallholder plantings in the municipality of Waslala, Nicaragua, were evaluated for two successive harvest periods. Cocoa samples were fermented using a technique mimicking recommended on-farm practices. The sensory cocoa quality was assessed by experienced tasters, and seven major chemical taste compounds were quantified by near infrared spectrometry (NIRS). The association of the nine, partially admixed, genotype spectra with the analytical and sensory quality parameters was tested. The individual parameters were analyzed as a function of the factors GG and harvest (including the date of fermentation), individual trees within a single GG were used as replications. In fermented cocoa, significant GG-specific differences were observed for methylxanthines, theobromine-to-caffeine (T/C) ratio, total fat, procyanidin B5 and epicatechin, as well as the sensory attributes global score, astringency, and dry fruit aroma, but differences related to harvest were also apparent. The potential cocoa yield was also highly determined by the individual GG, although there was significant tree-to-tree variation within every single GG. Non-fermented samples showed large harvest-to-harvest variation of their chemical composition, while differences between GG were insignificant. These results suggest that selection by the genetic background, represented here by groups of partially admixed genotype spectra, would be a useful strategy toward enhancing quality and yield of cocoa in Nicaragua. Selection by the GG within the local, genetically segregating populations of seed-propagated cacao, followed by clonal propagation of best-performing individuals of the selected GG could be a viable alternative to traditional propagation of cacao by seed from open pollination. Fast and gentle air-drying of the fermented beans and their permanent dry storage were an efficient and comparatively easy precondition for high cocoa quality.

## Introduction

The market of fine chocolates has been growing at the same pace as the overall mass chocolate market; it occupies constantly <5% of the global chocolate consumption. The finest qualities have been traditionally obtained using Criollo and Trinitario cocoas. Definitions of general trading grades of cocoa (bulk and fine) and morphogeographic types (Criollo, Forastero, Trinitario) were provided by [1] (pp. 29–36, 528–530). According to the International Cocoa Organization, the seven largest fine flavor cocoa producing countries were in 2010/11 Ecuador, Dominican Republic, Colombia, Venezuela, Madagascar, Nicaragua and Bolivia. The elevated prices paid for quality cocoa allow to enhance the incomes of poor smallholder producers that account for the bulk of global cocoa supplies. Increased revenues from cocoa also provide incentives for farmers to invest in crop management techniques toward increased sustainability of cacao plantations. However, the transition from the production of bulk cocoa to more differentiated and higher-value cocoa is, among other factors, hampered by the informal distribution of planting material without clear identity and performance guarantee, the general lack of appropriate selection of suitable genetic materials, and by the predominant use of botanical seed multiplication on-farm, which leads to much segregation of quality attributes.

In Nicaragua, the largest cocoa production area is in the municipality of Waslala [2]. These plantings have been established since 1981 using seed provided by the Tropical Agricultural Research and Education Center, CATIE (W. Phillips and C. Astorga, CATIE, pers. comm.). Comparative genetic analysis of the trees in Waslala and their ancestors at CATIE, Turrialba, Costa Rica, confirmed this [3]. In contrast, locally occurring ancient Mayan Criollo germplasm that survived in local, remnant rain forest was found unlikely to have contributed substantially to these present-day cacao plantings.

The present study was undertaken to 1) estimate the diversity of cocoa bean quality that can be obtained from Waslala, applying a standardized fermentation procedure and 2) test whether selection by the genetic background, such as determined by Trognitz et al. [3], can be used to increase quality and yield.

One hundred of 315 trees identified earlier [2] were used in the experiments. The quality of cocoa harvested from these trees in two periods, referred to as harvest 1 (from October 2008 until May 2009) and harvest 2 (November 2009–May 2010, with a few trees harvested as late as February 2011) was assessed by monitoring the quantity of several secondary metabolites and fat, key chemical taste compounds, by near infrared spectroscopy (NIRS) and sensory evaluations. These metabolites determine the specific value of cocoa as a stimulant food. The theobromine-to-caffeine (T/C) ratio, plotted against the caffeine contents was found to be a good indicator for quantifying the distinct qualities of the morphogeographic cocoa types, Criollo, Trinitario, and Forastero [4]. The richness of natural, unfermented cocoa in polyphenols, particularly procyanidins, correlates positively with an undesired astringent taste [5]. The procyanidins in the cocoa bean are reliably reduced by proper fermentation. Therefore, this postharvest processing step is the precondition for developing the final cocoa quality. Cocoa fat is sometimes seen as a means to transport and display cocoa’s flavoring agents in the mouth. Some manufacturers add, according to their preferences, up to 6% fat above the natural cocoa butter content present in cocoa liquor to achieve the optimal viscosity, mouthfeel, structure and taste of the chocolate, which should be brittle and shiny and fast-melting in the mouth. Therefore, a high fat content can facilitate chocolate production while reducing the need to use additional cocoa butter or other fat.

For the assessment of the relative amounts of the chemical compounds in dry matter through near infrared spectroscopy (NIRS), previously established, robust calibration curves were fitted, as is described in Materials and Methods.

## Results

### Analytics and Sensory Evaluation

During the first of two harvest periods (hereafter referred to as harvest 1), non-fermented and fermented samples were obtained from 95 of the in total 100 trees studied (six additional trees were included for assessment of potential yield only). In the second period (harvest 2), 87 non-fermented and 78 fermented cocoa samples were available for analysis, owing to the lack of suitable fruits from several study trees, and incomplete fermentation that led to the exclusion of some samples.

Fermentation considerably reduced both the methylxanthines theobromine and caffeine, and the phenolics (flavan-3-ols) epicatechin, procyanidins B2 (an (−)-epicatechin-(4β→8)-(−)-epicatechin dimer), B5 (an epicatechin-(4β → 6)-epicatechin dimer), and C1 (an epicatechin-(4β→8)-epicatechin–(4β→8)-epicatechin trimer). The average contents of total methylxanthines in the dry matter shrank from 1.505% of the dry matter (15.05 mg/g) in non-fermented to 1.294% (12.94 mg/g) in fermented samples and the four flavan-3-ols were diminished even more drastically from 3.408% to 0.697% (34.08 to 6.97 mg/g). The grand means of the compounds are given in Table 1, for a comparison of the contents in individual samples see Table S1.

**Table 1:** Summary of analyses of variance, average total quantities of chemical compounds, and sensory quality scores of cocoa samples.

Quality attribute	Df	GG	Harvest	GG×Harvest	Tree	Grand Mean	Min	Max
Fermented samples								
F Theobromine (T)	170	*	***	n.s.	**	1.04	0.54	1.49
F Caffeine (C)	170	***	***	n.s.	n.s.	0.254	0.05	0.5
F T/C	170	***	**	n.s.	n.s.	4.44	1.855	10.615
F Moisture	170	**	***	n.s.	n.s.	6.24	4.36	8.58
F Fat (6% moisture)	170	***	n.s.	n.s.	n.s.	58.50	52.7	63.9
F B2	170	n.s.	*	n.s.	**	0.111	0	0.24
F B5	170	***	n.s.	*	n.s.	0.032	0	0.15
F Epicatechin	170	**	n.s.	*	n.s.	0.369	0	1.38
F C1	170	n.s.	n.s.	n.s.	n.s.	0.185	0	0.4
Non-fermented samples								
N Theobromine	179	n.s.	***	n.s.	n.s.	1.238	0.21	1.73
N Caffeine	179	n.s.	***	n.s.	n.s.	0.267	0.07	0.51
N T/C	179	n.s.	n.s.	n.s.	n.s.	5.14	0.54	16.251
N Moisture	179	n.s.	***	n.s.	n.s.	7	5.08	12.38
N Fat (6% moisture)	179	n.s.	***	n.s.	n.s.	57.10	52.4	61.4
N B2	179	n.s.	***	n.s.	n.s.	0.405	0.1	0.57
N B5	179	n.s.	***	n.s.	n.s.	0.193	−0.02	0.35
N Epicatechin	179	n.s.	***	n.s.	n.s.	2.124	0.01	3.41
N C1	179	n.s.	***	n.s.	n.s.	0.686	0.13	0.98
NvF B5	170	n.s.	***	n.s.	n.s.			
NvF Epi	170	n.s.	***	n.s.	n.s.			
Sensory attributes, fermented and roasted samples (score 5; good, 0; bad)								
Aroma Intensity	172	n.s.	***	n.s.	n.s.	3.06	2	4.13
Global Score	172	**	***	**	n.s.	2.73	1.07	4.07
Acidity	172	n.s.	***	n.s.	n.s.	1.71	0.47	3.64
Bitterness	172	n.s.	n.s.	*	n.s.	2.88	1.71	3.93
Sweetness	172	n.s.	***	n.s.	n.s.	0.46	0	1.43
Astringency	172	*	***	n.s.	n.s.	2.08	0.93	3.4
Cacao flavour	172	n.s.	***	n.s.	n.s.	2.58	1.71	3.47
Fresh fruit	172	n.s.	n.s.	**	n.s.	0.98	0	2.2
Dry fruit	172	*	n.s.	n.s.	*	0.67	0	1.53
Unroasted seed	172	n.s.	***	*	*	0.83	0	2.21
Dry seed	172	n.s.	**	n.s.	n.s.	1.04	0.21	2
Floral aroma	172	n.s.	***	n.s.	*	0.67	0.07	2.73
Spiciness	172	n.s.	*	n.s.	n.s.	0.48	0	1.29
Wood (tannine) flavor	172	n.s.	***	n.s.	n.s.	0.56	0	1.5
Yield per tree	169	**	n.s.	n.s.	***	6.7 kg	2.7	14. 3

A quality attribute was considered as a function of Genotype Group (GG), Harvest, GG-Harvest interaction, and Trees (as replicates within a GG). The level of significance of variance components (F-test) is indicated (***; P<0.001, **; P<0.01, *; P<0.05, n.s.; not significant). There were 9 Genotype Groups (GG) and 95 (in harvest 1, non-fermented and fermented), 87 (harvest 2, non-fermented), and 78 (fermented) samples from individual trees. Df; error degrees of freedom (in analysis of variance). Grand mean quantities of compounds and their minima and maxima are presented in percent of dry matter.

In separate analyses of data from the two harvesting periods, normal distribution was rejected for the compounds procyanidin B5 and epicatechin in fermented samples (not shown). This was due to a large percentage of the fermented samples showing a strong reduction of these compounds relative to their non-fermented controls. However, there was no consistent pattern among the individuals. Trees whose fermented beans had, relative to the non-fermented samples, a much reduced B5 or epicatechin content in harvest 1, had no such reduction in harvest 2, and vice versa. Two composite traits were generated by subtracting the values of fermented from the values of the non-fermented samples {(B5 non-fermented) - (B5 fermented) and (epicatechin non-fermented) - (epicatechin fermented)}. Analyses of variance (using the model Trait = Tree replicated in Harvests) confirmed the absence of significant differences of these two traits between individual trees, whereas there were highly significant differences (P<0.001) between the two harvests (not shown). A major component subsumed under the factor Harvest represent the individual fermentations, which, due to restricted resources, could not be consistently replicated within a season for each tree’s fruits. Therefore, individual fermentations may contribute a major share of the variation of the Harvest factor.

**Figure 1:**
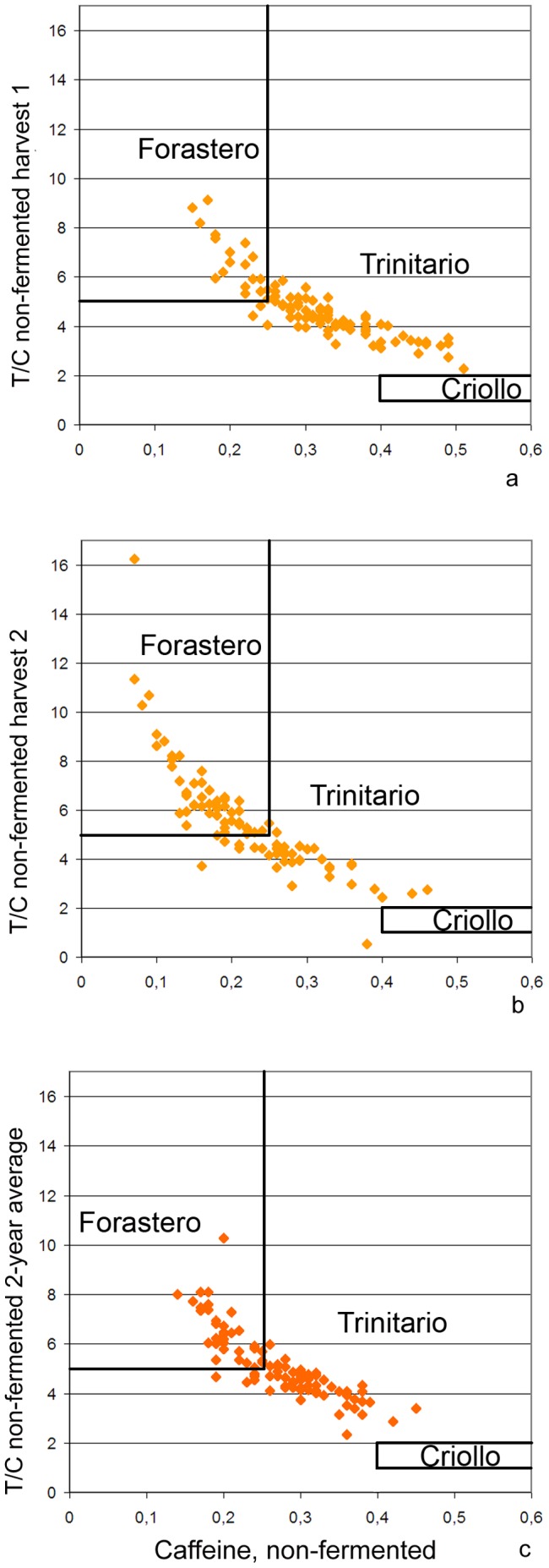
Plots of theobromine/caffeine (T/C) ratio (ordinate) vs. total caffeine content (abscissa) determined for samples of non-fermented cocoa. a; first harvest period 2008–9, b; second harvest (2009–11), c; two-harvest average.

Although several samples had high caffeine levels of 0.4–0.5% in harvest 1, none achieved a T/C ratio of 1–2 as is characteristic of ancient Criollo cocoa [4]. The majority (68%) of samples had an intermediate T/C ratio of >2 to <5 typical for Trinitario type cocoa, whereas a minor portion had T/C ratios >5 and caffeine contents <0.25%, as is characteristic for Forastero cocoa (Figure 1a). In harvest 2, the caffeine content was generally more reduced in the samples (Figure 1b), and averaged over the two harvests (Figure 1c), most trees had methylxanthine contents typical of Trinitario cocoa.

### Performance of Individual Cacao Trees

Table S1 gives the contents of chemical compounds in fermented and non-fermented samples, scores of sensory attributes, and potential yield for every individual tree, averaged over two harvests. There were large differences between individuals, including the contents of theobromine, caffeine and major phenolics, as well as the global score of the sensory performance. However, almost all these characteristics showed highly significant (P<0.001) harvest-dependent variation, as was revealed in analyses of variance using Tree as a factor and Harvest as replication term (not shown). Significant, exclusively tree-related characteristics independent of seasonal influences were fat contents of fermented beans (P<0.001) and the bitterness score (P<0.01). Although caffeine, theobromine, B2, and C1 levels of fermented samples, as well as the global score and floral flavor intensity score also had significant tree-related variation (P<0.01–0.001), they heavily depended at the same time on the individual harvest (P<0.001).

### Assessing the Yield Potential

Yield estimates were available for 93 trees in the first and 90 in the second period of experimental harvests. The average potential (theoretical) cocoa yield of these elite trees was calculated at around 6.4–6.7 kg ranging from 2.7 to 14.3 kg/tree. This is equivalent to 130 fruits from an average tree, with 39 fully developed seeds per fruit and a 1000-seed mass of 1280 g (air dried, <8% water content). Separate analyses of variance in general linear models revealed significant dependence of the yield on the individual tree (replicated in 2 harvests; P<0.001, harvest-related changes were marginally significant at P<0.05), the individual genotype group (trees representing replicates; P<0.01) and the individual farm (with a farm’s trees as replicates; P<0.001). The significance of factor Farm was likely caused, besides by individual farmers’ practices, by the trees growing within a single farm being genetically closely related due to the prevailing method of seed multiplication through uncontrolled pollination including an unknown portion of selfing.

### Associations among Attributes

**Figure 2:**
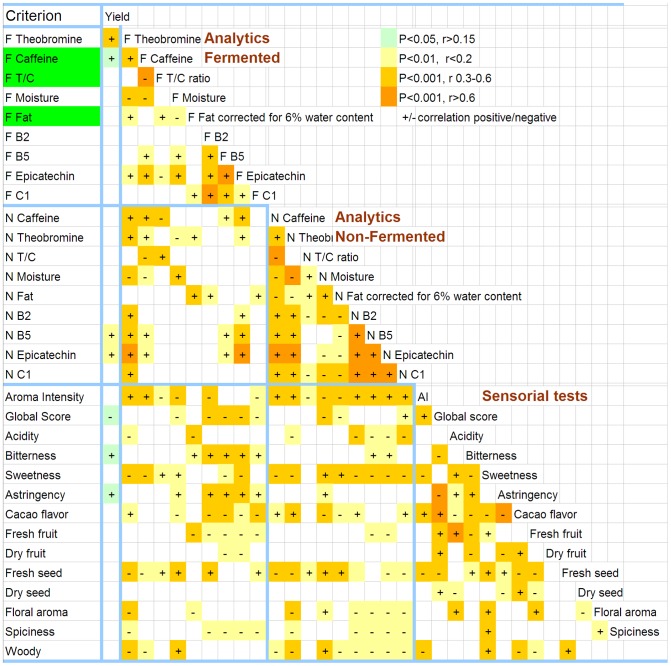
Pairwise correlations of analytical (F; fermented and N; non-fermented samples) and sensory data of fermented samples obtained on up to 172 individual observations collected over two harvests of cocoa from 100 trees of smallholder farms around Waslala, Nicaragua. Color and shading intensity indicate levels of significance of correlations. Theobromine/caffeine (T/C) ratio and theobromine and fat contents of fermented cocoa (highlighted by green background) were the most important characteristics as determined in the analytics (see text).

Pairwise Pearson’s correlations among the analytical and sensory traits (Figure 2) revealed the most significant associations between chemically closely related items within each of the groups of fermented and non-fermented samples and between the items of the sensory evaluation complex. Caffeine and theobromine contents were correlated for both fermented and non-fermented samples. Theobromine contents were correlated with the phenolics across non-fermented, but not across fermented samples. The two methylxanthines were also positively correlated with the sensory attribute aroma intensity (AI) and negatively with sweetness and taste of unroasted nut/seed (fresh almonds, hazelnuts). Interestingly, AI was correlated with all chemical compounds in non-fermented cocoa (r = 0.3 to 0.6, P<0.001), whereas with the compounds in fermented samples only six of all nine pairwise correlations were significant. A similar decline of associations can be stated for the sweetness score. In other words, the quantities of the chemical compounds in non-fermented cocoa beans might be a good indicator of several sensory attributes that become manifest in fermented and roasted cocoa. Several sensory attributes correlated either directly or inversely with the analytics data in fermented cocoa, but these correlations tended to be weak (r<0.2, P<0.01). An exception was the positive correlation of bitterness and astringency with the contents of the phenolics in fermented samples; correlation coefficients were between 0.3 and 0.6 and the confidence level was above 0.99 (P<0.001). In contrast, the correlation of bitterness and astringency scores with the quantity of phenolics in non-fermented samples was mainly absent. Among the parameters of the sensory evaluation, positive correlations (r = 0.3 to 0.6, P<0.001) occurred between bitterness and astringency. The acidity score was positively correlated with sweetness, fresh fruit taste and floral aroma scores.

Elevated levels of residual moisture were associated with markedly reduced AI and global score, while the sensation of woody and tastes of unroasted nut/seed (fresh almonds, hazelnuts) increased. This underlines the importance of fast drying of the cocoa beans and their storage under dry conditions.

### Association between Biochemical and Sensory Phenotype and Genetic Background

**Figure 3:**
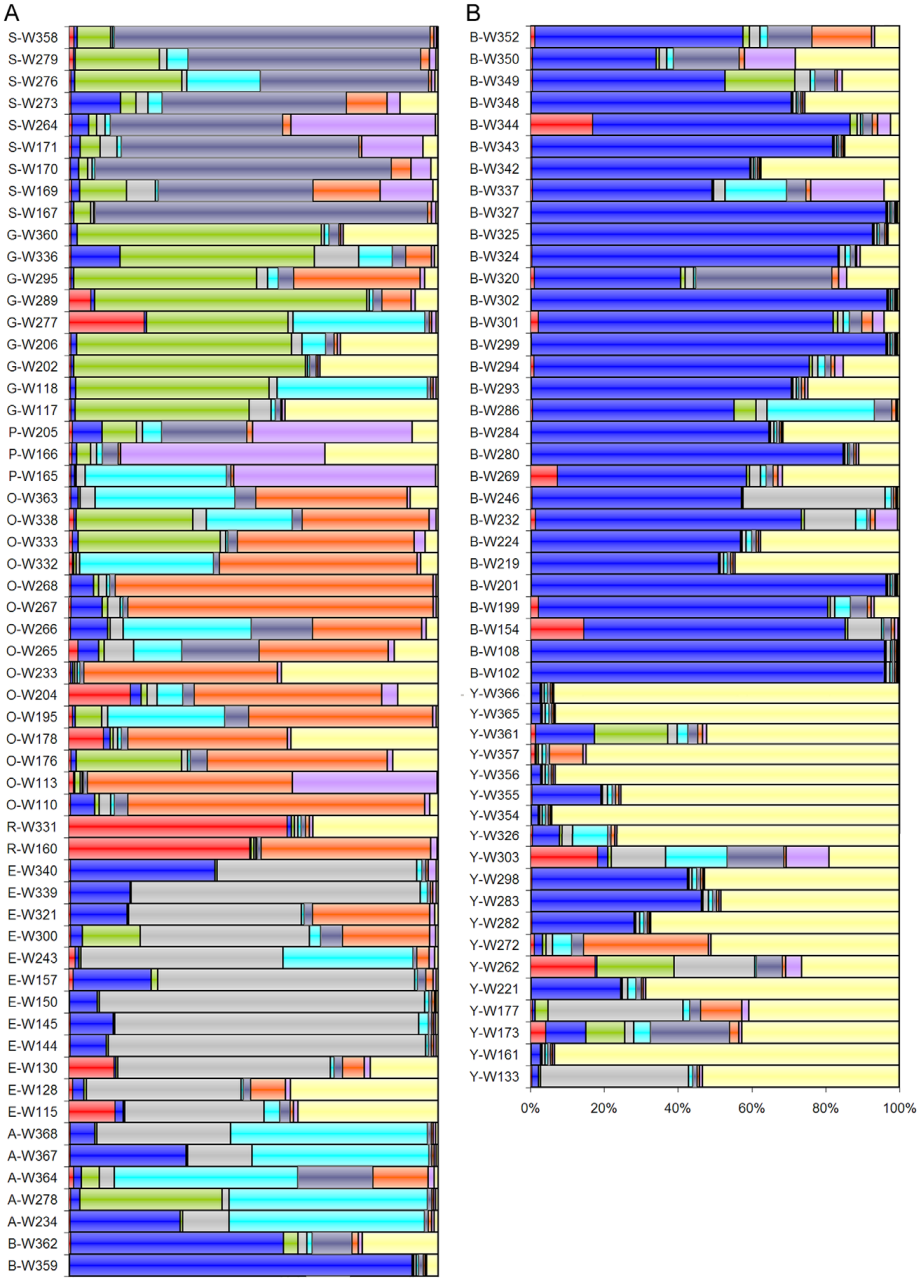
Bar plots of the 15-SSR-genotype of 106 cacao trees from farms at Waslala that were used for assessment of cocoa quality and yield potential. A single bar represents a tree’s cumulative genotype consisting of fractions of ancestral genotype spectra. The in total nine genotype spectra were determined in [3] via Bayesian inference of population structure. Color coding of individual genotype spectra according to [3]. S; steel gray, G; green, P; purple, O; orange, R; red, E; gray, A; aqua, B; blue, Y; yellow. Their most probable ancestors and traditional cocoa types include for S; lower Amazon Forastero, G, P, O, and R; upper Amazon Forastero, E; upper Amazon Forastero isolated in coastal Ecuador, A, B; Trinitario, and Y; Criollo from Mesoamerica. The trees were grouped by their dominating genotype spectra. Group names of genotypes are indicated prior to tree name (as an example, tree W358 belongs to the S genotype group).

The genotype of a single tree was defined as the specific combination of genotype spectra (GS) obtained by inference of population structure using the program Structure [6], [7], as described in Trognitz et al. [3]. Because most trees were hybrids combining several admixed GS they were assigned to nine groups of genotypes (GG), according to the GS that had the greatest share of their genotype (Figure 3). Analyses of variance (ANOVA) were carried out for every analytical and sensory attribute as the dependent variable, treating GG and Harvest as independent factors. Individual trees within a single GG were considered as replicates (error term). Table 1 shows the degrees of significance of the variance components (factors), as determined by F tests. In general, the parameters for fermented cocoa were significantly influenced by the GG, although variation by Harvest also was significant for several characteristics. An exception was fat content; it was solely determined by GG. The phenolics also were much dependent on GG, with marginal variation due to the individual harvest or tree. Interestingly, no association of GG was apparent for any attribute in non-fermented cocoa, whereas Harvest always was a highly significant determinant of the performance, thereby confirming the importance of individual conditions of post-harvest treatment. Likewise, the sensory test results were strongly influenced by Harvest. An exception was the dry fruit flavor that appeared to be marginally controlled by GG and Tree (P<0.05), however, the generally low scores given in the evaluation (average, 0.67 on a scale of 0–5) rendered this trait less useful for assessing cocoa quality. The global score, which is a holistic summary of taste and aroma components based on the experience of the individual evaluators, was somewhat depending upon the GG, but again, it showed stronger dependence on the individual harvest.

### Yield and Genetic Background

The potential yield was significantly under the control of GG (Table 1), with large tree-to-tree differences that resulted in reduced differentiation of GGs by their average yield levels (Table 2, overlapping groups A, B and C). Table 3 shows for all nine individual genetic backgrounds detected earlier [3] the average scores of those chemical, sensory, and yield characteristics that were, according to F tests in ANOVA, significantly controlled by the GS (represented by GG).

**Table 2:** Potential cocoa yield (<8% water content) of nine Genotype Groups (GG) and their ranking and differentiation by comparison of multiple means (t-test).

GG^1^	Samplings	# Trees	Yield (kg)	t-Test
S	14	9	7.4	A^1^
R	4	2	6.9	AB
B	51	32	6.9	AB
Y	32	19	6.7	AB
P	4	3	6.5	AB
O	28	15	6.0	B
G	15	9	5.8	B
A	9	5	5.1	CB
E	12	12	4.6	C

1 GG; Genotype Group, compare Figure 3, Samplings; Number of observations made on the trees. The objective of 2 samplings per tree was not achieved for all trees. # Trees; Number of trees within a GG, Yield; Least-squares mean potential yield per tree (in kg). The potential maximum single-tree yield was estimated as (no fruits past cherelle wilt stage) * (no beans/fruit) * (bean weight). Bean weight was averaged on 100 dry (<8% water content) beans. t-Test; least significant difference of yield by GG determined by multiple t-Tests. Values with identical letters are not significantly different.

**Table 3:** Fermented cocoa from 100 individual trees (genotypes) representing 9 cacao Genotype Groups. Relative amounts of chemical compounds and scores of sensory attributes.

		Theobromine	Caffeine	T/C	Fat	B5	Epicat	Global	Dry Fruit	
N	GG	Analytics						Sensory evaluation		Yield
32	B	1.06	0.29	3.93	57.7	0.04	0.47	2.73	0.68	
19	Y	1.04	0.29	3.81	58.9	0.04	0.52	2.52	0.53	
15	O	1.05	0.23	4.9	59.2	0.02	0.24	2.81	0.73	
12	E	0.98	0.24	4.26	57.5	0.03	0.38	2.66	0.66	
9	G	1.09	0.26	4.28	59.1	0.02	0.25	2.87	0.66	
9	S	1.08	0.22	5.49	60.2	0.02	0.26	2.89	0.83	
5	A	0.99	0.18	5.65	59.7	0.04	0.48	2.47	0.44	
3	P	1.03	0.18	6.31	56.9	0.06	0.5	2.87	0.68	
2	R	1.06	0.26	4.17	59.3	0.05	0.47	2.68	0.69	
	**Rank**	**Genotype group**								
	1	**G**	**B**	**Y**	**S**	**P**	**Y**	**S**	**S**	**S**
	2	**S**	**Y**	**B**	**A**	**R**	**P**	**P**	**O**	**R**
	3	**B**	**G**	**R**	**R**	**A**	**A**	**G**	**R**	**B**
	4	R	R	E	O	Y	R	O	P	Y
	5	O	E	G	G	B	B	B	B	P
	6	Y	O	O	Y	E	E	R	G	O
	7	P	S	S	B	S	S	E	E	G
	8	A	P	A	E	O	G	Y	Y	A
	9	E	A	P	P	G	O	A	A	E
	P(F)	*	***	***	***	***	**	**	*	**

Two-harvest averages by Genotype Group (GG) are indicated in the top part. GGs coded by colors according to [3] (B; blue, Y; yellow, O; orange, E; gray, G; green, S; steel gray, A; aqua, P; purple, R; red).

Bottom part of table; individual GGs are ranked for each character (in descending order from positive to negative contribution to high quality cocoa). The probability P (*; <0.05, **; <0.01, ***; <0.001) of a character as under control by the GG is indicated in the last line. To decide which GG represents the greatest potential (as a decision support in selection) one could consider those that occur most frequently among the (highlighted in bold-face) top three ranks. These would be GGs S and R, both occur 5 times.

The influence of the factor Farm was also tested, and certain yield levels were associated with individual farms. In the ANOVA of the model Yield = Farm+Harvest+Farm×Harvest+Tree (as within-Farm replicates), only Farm was significant (P<0.001). Farm-related variance was weakly or not at all significant for several biochemical and sensory characteristics, and when the few farms represented by only a single tree were removed from the data, Farm was uniformly insignificant. Therefore, associations of Farm with attributes other than yield were considered spurious (not shown).

## Discussion

### General Quality Characteristics of the Cocoa from Waslala

Figure 1 suggests the cocoas of Waslala can be considered as Trinitario, despite their great variation. Our plots of T/C ratio vs. absolute C (caffeine) contents were a useful means to visualize the discrimination of the commercial varieties Forastero, Trinitario and Criollo. This graphical method takes into account both the quality determining ratio of the methylxanthine alkaloids and their absolute quantity. In particular, high levels of caffeine are characteristic of the high quality Criollo cocoas, as was determined by investigating a large number of reference samples representing these distinct qualities [4] throughout many years. The T/C–C plots of cocoas from Waslala indicated as expected that they correspond to Trinitario. The attributes constituting cocoa quality varied greatly among individual trees. The chemical analytics of fermented samples appeared most appropriate for a solid assessment of quality. Total fat content was the most reproducible of all characteristics studied, despite the fairly small absolute differences between individual trees and genotype groups (Table 3, group average min; 57.5%, max; 60.2%). Fermented cocoa showed significant GG-related effects on the contents of theobromine and caffeine, as well as epicatechin and its dimer, B5 (Table 1), whereas for non-fermented samples no GG effects were evident. This contrast between fermented and non-fermented cocoa highlights the significance of proper fermentation for the production of chocolate. Important characteristics defining the quality of cocoa are ultimately generated during the fermentation and roasting process [8], [9]. These effects seen in the analyses of variance are not to be confounded with the effects of the genetic diversity per se. There is tremendous genetic variation within the individual farms of Waslala (compare [2]) and not even the best fermentation conditions can produce high quality cocoa of a tree that is by its genotype predetermined to produce low-quality cocoa.

Of all traits investigated, the most stable was total fat contents, which showed insignificant seasonal (harvest- and fermentation-related) effects throughout unfermented and fermented samples. In contrast, several samples lost large parts of their polyphenols during fermentation. This phenomenon was observed in both harvests, but there was no pattern; samples from different trees were affected indicating the influence of non-controlled phenomena, such as specific, unknown conditions related to individual fermentations. Elwers [8] also observed a dramatic reduction of the polyphenol content during fermentation, and she discussed genotypic peculiarities as potential sources of this variation. In particular, she hypothesized that the Criollo type would change during fermentation due to distinct anatomical features of its seeds (peculiar permeability of the testa, specific properties of the adhering pulp, efficient polyphenol oxidases). As an alternative reason, potentially genotype-specific turnover rates of the phenolics, independent of fermentation, were suggested [9]. We have observed that the round shape of Criollo seeds may facilitate the aeration during the fermentation, whereas Forastero possesses somewhat angular seeds that get tightly stacked and thus limit aeration. However, as a more plausible reason, our samples may have contained varying quantities of differently mature seeds and thus varying richness in phenolics. Within the 2–3 weeks near the final stages of fruit maturation, dramatic changes in concentration of the phenolics were reported [8], [10]–[12]. However, in external, easily recognized characteristics these indicators of maturity become only scarcely manifest. The fruits of most genotypes change their color, and this alteration takes several days that are important for maturation. The testa of the seeds turns pink already before maturity, and color is no reliable marker of maturity [8], [9]. The inconsistent reduction of B5 and epicatechin during fermentation observed in our experiment corroborates these earlier findings [8]–[12] on the difficulty to determine in the field the optimal stage of cocoa seed maturity for processing purposes and to control the conditions of fermentation. The issue of fast and gentle drying after fermentation and dry storage is of greatest relevance. As can be seen from Figure 2, the higher moisture levels (of fermented samples that were found least dry at the time of analysis) were negatively correlated with the desired sensory traits AI and Global score and positively correlated with the undesired taste of unroasted nut/seed (fresh almonds, hazelnuts) and woody tastes. The higher the water contents of the raw cocoa is the more quality deductions have to be expected. For the preservation of high quality, speedy and gentle (avoiding direct solar radiation and burning through excess heat) drying and maintaining permanently dry storage conditions throughout the entire production and market chain is one of the simplest and most efficient measures to take.

### Ways to Assess and Characterize the Quality of Cocoa

Aroma and taste are the key factors determining the potential quality of cocoa. In our investigation (Table 1) aroma and taste frequently depended on the specific environmental conditions (harvests including fermentations) and not only on the genotype. Although the general cocoa quality (assessed here as the global score) showed association with the individual genotype group (P<0.01, Table 1), its dependence on individual harvests, including fermentations (P<0.001) was much greater. Other sensory characteristics appeared even more depending on individual harvests and fermentations and less on the genotype. For the purpose of selection in breeding, the complex evaluation of aroma and taste requiring expensive replications across many harvests, fermentations and years is only conditionally applicable under the prevailing cocoa production on small subsistence farms. High positive correlations amongst sensory attributes were observed between cocoa flavor and global score as well as between fresh fruit and acidity (Figure 2). This suggests that the trained tasters may perceive cocoa with a pronounced basic cocoa flavor as preferable and thus, give a high overall quality score to such samples. The association of fresh fruit with acidity indicates that both variables are highly interdependent or difficult to differentiate. Therefore, the identification of taste and aroma attributes, besides the improvisational global score applied here, with high genetic heritability that could be assessed in standardized organoleptic tests still remains pending.

In contrast, chemical analytics revealed compounds whose levels were reproducibly associated with the quality of cocoa (Table 1). In particular, total fat contents and the amount of procyanidin B5 and epicatechin in the fermented beans were excellent attributes with strong relation to the genotype. Theobromine and caffeine contents and T/C ratio were less useful characteristics due to their large harvest-related variation. Given the close correlation of the characteristics within their chemical classes (Figure 2) it might be sufficient for successful selection to use one compound out of the methylxanthines, such as theobromine, and one of the flavan-3-ols, for example the pro-epicatechin B5, as quality markers.

The comparatively strong association among increasing contents of the methylxanthines (particularly theobromine, whose concentration in the samples was the 4.1–fold of caffeine) and aroma intensity highlights the importance of these alkaloids for the quality of cocoa. Our results are in line with the work by [5] who identified 84 taste-determining cocoa compounds where theobromine was confirmed as the prominent determinant of bitterness. Although our study involved only a small fraction of the compounds investigated by [5] it is interesting that the bitter alkaloids contents showed such a great association with what we considered aroma intensity of cocoa. Theobromine was also detected earlier as the bitterness-attributing compound when it is combined with diketopiperazines, which are generated as thermic decay products during the roasting of cocoa [13].

Epicatechin and procyanidins add to an astringent and lip puckering sensation [5], and the quantity of these compounds was positively correlated with the astringency and bitterness scores assigned by our panel of tasters (Figure 2). The association of sensory attributes with the concentration of chemical compounds was apparent in fermented samples only. The concentrations of procyanidins in fermented and non-fermented samples were only partially correlated (Figure 2).

### Association of Chemical and Sensory Attributes and Potential Yield with the Genotype

The future of cocoa enhancement increasingly depends on the application of molecular genetics. Already our comparatively rough 15-SSR-fingerprint genotype had predictive power for the quality phenotype of fermented, but not of non-fermented, cocoa. Specifically, the total fat, procyanidin B5 and epicatechin contents were under the control of genotype group (Table 1). There was still very large variation caused by individual trees (unique, only partially similar genotypes within a genotype group) and harvests (environment, including fermentations). But we are confident that further refinement of genotyping through the use of more specific molecular markers soon will substantially increase the predictive power in cacao selection by the genotype. A system at the hand of breeders, such as the synopsis of parameters proposed in Table 3 and Figure 3 can then be used to support decision-making in selection.

To demonstrate the disadvantages of the continued dependence in selection on traditional, morphological traits, we have recorded the cacao fruit type as it was assigned by the farmers. This type reflects the shape, color, and appearance of the fruit surface and is highly heritable (Table S1, compare also [2]). No association of fruit type with any chemical or sensory characteristics of the cocoa was apparent, suggesting that selecting for quality cannot rely on the few variable morphological features of the cacao tree, and this emphasizes the importance of making reference to the genotype as a viable alternative.

### Technological Means to Achieve High-quality Cocoa

The apparent great variation of cocoa quality due to the individual harvest and fermentation underlines the importance of these ‘environmental’ factors, even within this comparatively narrow production area in Nicaragua. We have observed considerable quality differences for cocoa produced either in the Republic of Côte d’Ivoire or in the Republic of Vanuatu from clonally propagated trees of individual accessions. However, there are a few distinct flavors that apparently are expressed in many environments. One example is the distinct flavor of accession SCA6 that was invariably evident in cocoa samples from Brazil, Republic of Côte d’Ivoire, and Malaysia (EC, SA, unpublished data).

In general, strict adherence to harvesting perfectly mature fruits, adequate fermenting, careful drying and dry storage, and other adequate postharvest treatment is of utmost importance for the constant production of high-quality cocoa. At Waslala, most trees were able to produce cocoa of very acceptable global scores and pronounced cacao taste, as was obtained in at least one harvest. One in three to four trees tested gave cocoa of excellent individual sensory attributes ranging from floral over sweet to fruity notes. There is no doubt that the best-performing trees, as judged on the two-harvest average global note, together with some trees that exhibited specific floral and spicy attributes, are worth to be propagated clonally and distributed across Waslala, with the objective of establishing local planting stocks as a precondition for achieving consistently high and distinct local cocoa quality.

### Assessing the Yield Potential of the Cacao Trees in Waslala

Statistics of the CACAONICA cooperative and its partner organization, Pro Mundo Humano, indicate an average triennial yield (until 2007) of dry fermented cocoa of 328 (175–452) kg/ha (CATIE, unpublished data) achieved at small plantings (0.75–1.5 ha) with a density of 750 cacao trees/ha (H. Grebe, Pro Mundo Humano, Waslala, Nicaragua, 2011, pers. comm.). For a preliminary estimation of the potential maximum yield, we used number of fruits on a tree past the cherelle wilt stage, number of beans per fruit, and dry weight per bean. Losses due to disease, predation, and mechanical damage represented 10–25% on a tree basis (EA, unpublished results). Considering the average potential yield estimated for our elite trees (Table 2 and Table S1) and the density of 750 trees/ha, this would amount to a yield of 3.75–4.5 t/ha. However, in practice, only a fraction of the potential yield as calculated here is obtained. One determinant of the yield potential was the genetic background (Table 1). Therefore, this 15-SSR fingerprint information will be valuable when selecting for elevated levels of yield potential. The observed, significant association of individual farms with particularly high, or low, levels of yield was not surprising, as the individual farms accumulated trees of specific genetic backgrounds, as a result of the predominant sexual propagation by seed from trees already present and an uneven initial distribution of germplasm at the time when the farms were established [2], [3].

### Conclusions

In this study of the cocoa from 100 cacao trees representing the nine distinct genotype spectra, which make up the plantings in Waslala, Nicaragua, it was found that even the relatively gross 15-SSR fingerprints used were sufficient to reveal genotype-related, and thus heritable, differentiation of several traits (Table 1). Among these, total fat contents of fermented beans had the strongest association with the genetic background, while variation between the two harvests (including individual fermentations) was insignificant. In addition, the contents of epicatechin and procyanidin B5 in fermented beans was under significant control by GG (Table 1, upper part).

Theobromine and caffeine concentration, although also significantly determined by the genetic background (GG), were modified to a large part by the different harvests (and fermentations). In non-fermented cocoa, none of these characters was associated with the individual genotype groups (Table 1, center part). This is probably due to the large tree-to-tree variation within a genotype group that was necessarily created by investigating seed-propagated individuals (tree genotypes possessing different combinations of identical alleles) grown at a specific farm location. Additional studies on fewer, selected genotype groups that are clonally replicated and thus avoid the great genetic variability of generatively propagated trees will help to elucidate the contribution of the genotype to these characteristics.

The current observations underline the great importance of fermentation for the production of cocoa. Several compounds are modified or even formed during the various stages of this complex process when the temperature rises and the disrupted cell membranes allow compounds of the storage and pigment cells to diffuse. The difference between the genetic backgrounds (GG) was not the contents of specific compounds per se, but the individual response to fermentation.

The majority of attributes estimated in sensory tests depended on the individual harvest and fermentation, with the exception of the dry fruit taste. The good correlation among several attributes allows the reduction of their number in future investigations, without much loss of information. Nonetheless, systematic, yearly sensory evaluations on-site may be helpful to detect individual trees that can produce cocoa of distinct culinary quality.

For the purpose of enhancing a production area’s cocoa quality and yield, selection based on the genotype will be a reliable, reproducible, and secure method for sustained progress in breeding. The current 15-SSR-marker fingerprint is a baseline that can be extended by adding quality-related molecular markers. The identification of distinct genotype groups within the pool of Trinitario cacao provides ample room for selection within elite groups among individual trees based on agronomic characteristics. Such a methodology will circumvent the problems inherent in a monoculture crop resulting from the clonal mass-propagation of only one single individual.

Taking into account the best-performing genetic backgrounds, quality attributes, and yield potential as determined in this study, twenty most promising individuals among the 100 genetically characterized trees have been selected and are being clonally (graft-) propagated for distribution among Waslala’s smallholder farms. These materials will also serve as a base for subsequent studies toward the establishment of plantings with enhanced cocoa quality and yield under improved crop management conditions (including directed pollination). Further project work will be aimed at determining these conditions. We are confident that the traditional rule of thumb of only 30 percent of all trees contributing 70 percent of a farm’s cocoa can be overcome with more productive cacao plantings that have a much greater share of high-performance trees.

Finally, the big gap between the actual yield of about 0.33 t/ha currently achieved in Waslala and the potential yield level of 5 t/ha, as was inferred from a limited number of high-yielding trees, illustrates the potential for improvement based on local genetic resources. Exploring the causes that underlie the high yield of the selected elite trees and applying the lessons learned could be a first step toward enhancing and stabilizing the cocoa yield under the conditions of the existing farms.

## Materials and Methods

### Cacao Trees and Genetic Fingerprints

One hundred trees from smallholder plantings near the town of Waslala, Nicaragua, were chosen from the 317 trees investigated earlier [2], [3]. The 100 trees are evenly distributed across 31 plantings covering all 14 agro-climatic zones in the Waslala municipality as determined previously [2]. Additional criteria for the choice of the trees were high yield, as indicated by the owners, accessibility by the project staff for monitoring fruit production, and ease and speed of transport of mature fruits to the central processing site. Information on these trees’ 15-simple-sequence-repeat (SSR) marker genotypes (assembled using the SSR primers indicated in [2]) was extracted from the result of data analyses in [3]. There, it was detected by inference of population structure using the Bayesian approach implemented in the program Structure [6], [7] that the genotype of every individual tree consisted of parts of up to nine distinct genotype spectra.

All necessary permits were obtained for the described field studies. The cocoa cooperative CACAONICA, Waslala, Nicaragua, and its associate farmers who provided trees for the study, acted as participants in the research and development project entitled ‘Sustainable futures for indigenous smallholders in Nicaragua: harnessing the high-value potential of native cacao diversity’. This publication describes results from the project. The project aims at assisting farmers in Waslala to make a gradual transition from their current production of only average quality cocoa to the production of differentiated high value cocoa based on the optimized use of cacao biodiversity, choice of locality and appropriate post-harvest procedures. For the shipment of dry leaf samples (dried on silica gel to <5% relative moisture), phytosanitary permits were granted by the Nicaraguan ministry of agriculture and forestry (Cetrex-FUEMA 118091-1-FI4WV8, 156882-1-FIRST6, 196133-1-FIBZY6T) and by the authority of Costa Rica (permit No. 1061838).

### Harvest and Processing of Cocoa Samples

**Figure 4:**
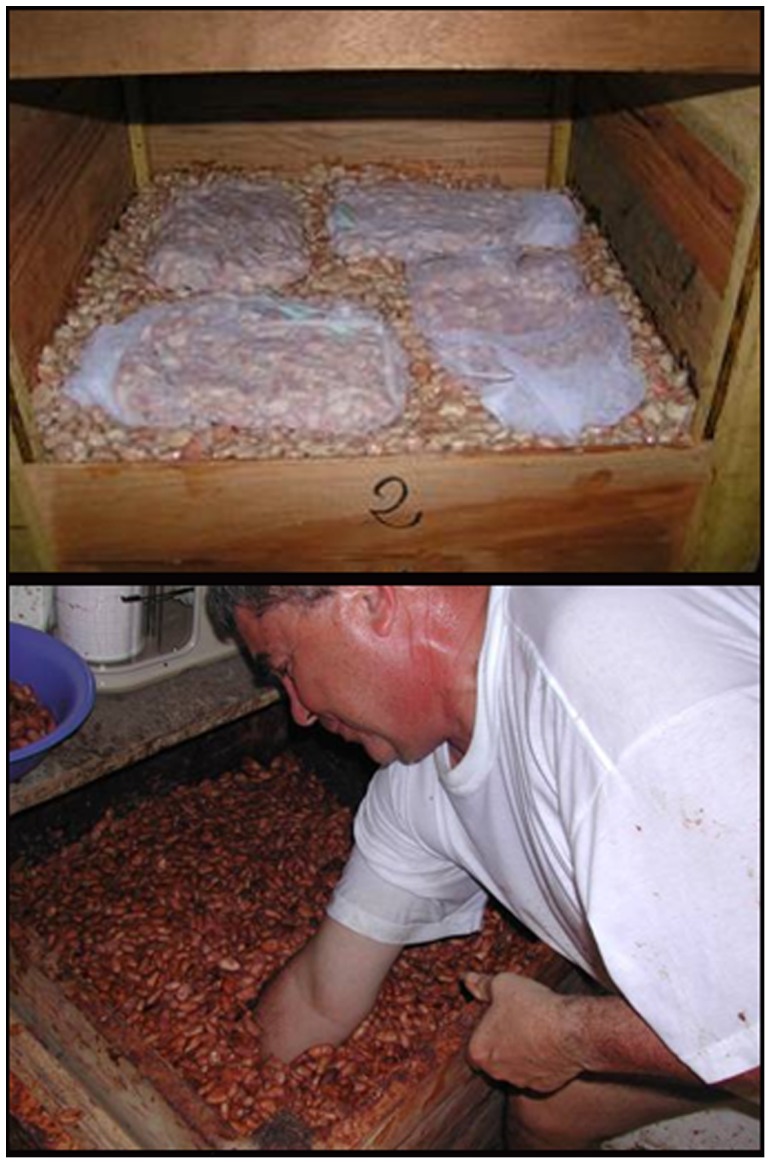
Method of fermenting several small cocoa bean samples from individual trees under identical conditions of recommended traditional processing practice (“microfermentation technique”). A wooden fermentation box of 0.6×0.6×0.6-m dimensions is filled with a uniform 180-l batch of fresh beans. Up to eight meshes containing the individual samples are evenly distributed across two layers and perfectly embedded in the batch beans. Banana leaf is added as a cover and to provide optimal conditions for fermenting bacteria. The entire volume is rearranged manually every second day. Fermentation time uniformly was six days.

Fully mature, healthy fruits were harvested from November 2008 to May 2009 (first replicate) and from November 2009 to May 2010 (second replicate, several samples were collected until February 2011), when enough mature fruits could be obtained simultaneously from a tree to yield typically 1.3 kg, but at least 1 kg (wet weight) of seeds (including tightly adherent residues of the aril) per tree. Entire, healthy fruits of a farm, including the harvest of the experimental trees (separately bagged and properly labelled) and also of many other trees within this farm, were transported to the project’s own central processing (fermenting) site in Waslala in the afternoon of the day preceding the onset of the fermentation treatment. In the following morning, seed (beans surrounded by the adhering pulp) of the experimental trees were removed from the fruits, mixed and distributed into two separate, labelled samples. One sample of ca. 100 g was immediately set to dry (unfermented beans), while the larger second sample (ca. 1200 g) was placed in a nylon mesh coarse enough to allow for the percolation of viscous fermentation fluid. The seeds of these trees exceeding the 1.3 kg required for investigation together with the seeds of that farm’s other trees were pooled to provide a batch of nearly 200 l for fermentation in a single box. Up to eight bags containing samples of individual experimental trees were embedded in the batch of pooled beans in a wooden fermenting box of 0.2 m^3^ volume (0.6×0.6×0.6 m) made of untreated wood, as is frequently recommended for commercial processing (compare Figure 4). In detail, the bottom of a fermenting box was covered with 10 cm of wet bulked beans. A first layer of 4 sample bags was placed at a distance of about 5 cm between adjacent bags and the borders of the box. A 10-cm layer of bulked beans was added and then a second layer of 4 samples, which was covered in turn by another 10 cm bulk beans. The height of the entire heap within a box was 50 cm, this was sealed with banana leaves and the wood cover of the box. Typically, the harvests of 2–4 farms were processed and fermented (in 2–4 boxes, accordingly) simultaneously. The boxes were uniformly incubated for 6 days with rearrangement of the bags and thorough mixing of the surrounding bulk beans after 48 and 96 hours to ensure homogeneous and complete fermentation.

Both non-fermented and fermented samples were gently dried to <8% water content at ambient air on wooden tables and movable roofing to protect from rain and extreme sun.

Two identical dryers (flat wooden crates, divided by boards into 25×25-cm-compartments), which can be easily hand-carried, were built to dry the cocoa samples. The samples of the unfermented beans were immediately deposited within these compartments, the fermented samples were dried after the 6-day fermentation. On the first day, the dryers were placed under light shade and the beans were mixed every 2 hours. On the subsequent days the dryers were placed under sunlight for 2 hours, then for 4 hours and finally for the entire day while the two-hour intervals of mixing the beans within the samples and permuting the placement of the bagged samples were maintained. During the nights the dryers were stored in a closed room.

The mass of a sample from a single tree was 500 g of fermented and 50 g of non-fermented, dry (<8% water content) cocoa beans. All dried cocoa samples were stored at −14°C until shipping (by courier) to the cocoa quality laboratory at CIRAD, Montpellier, France, for further processing and investigation. There, prior to elaboration of a sample for analytical or organoleptic testing, malformed, diseased, and damaged beans were removed.

### Cocoa Analytics

Intact and visually perfect beans of non-fermented and fermented (without roasting) samples were carefully manually shelled and coarsely ground in a Limprimita cocoa breaker (Capco, Ipswich, UK) and then ground further in a knife grinder in liquid nitrogen to avoid heating transformations, until obtaining a maximum 0.5 mm particle size. The resulting cocoa powder was sieved (0.5 mm) and kept at −80°C until analysis in a FOSS 6500 near infrared spectrometer (Welltech, Capitol Heights, ML, USA). Of 3-g cocoa powder samples delivered in 50-mm diameter ring cups, reflectance was scanned at 2-nm intervals, from 400 to 2500 nm. The data (average of 32 scans per wave length interval) was stored as log(1/R) with R being the reflectance and 1 referring to the reflectance of a standard ceramic reference. The spectrum acquisitions were at random. Each sample was measured twice. Statistical analyses were made using Win-ISI II (Infrasoft International, Port Matilda, PA, U.S.A.) and XLStat (Addinsoft, Paris, France) software. The spectra were mathematically corrected for light scattering using the standard normal variate and Barnes detrend correction [14]. Second derivatives were calculated on five datapoints and smoothed using Savitzky and Golay polynomial smoothing [15] on five data points. The relative contents (in percent of total dry matter) of total fat and dry matter, the methylxanthines caffeine and theobromine, the flavan-3-ol epicatechin and the procyanidins B2, B5, C1 were assessed based on calibration curves developed at CIRAD (Table 4, note that the values reported may therefore divert from values determined following the newly developed AOAC guidelines [16]). For each sample, the average of two replicated measurements was recorded. The theobromine-to-caffeine (T/C) ratio was plotted against the total caffeine content of non-fermented samples (according to [17]) and used as a characteristic for similarity to the classical cocoa types, Criollo (T/C 1–2, caffeine 0.4–0.8%), Forastero (T/C 5–14, caffeine 0.1–0.25%), and Trinitario, intermediate to Criollo and Forastero [4].

**Table 4:** Empirically determined mean values (expressed as percent of air-dried cocoa matter) and their statistics, generated and used at CIRAD to predict the quantity of several biochemical compounds in cocoa samples via Near Infrared Spectroscopy (NIRS).

Compound	n	Mean	SD	SE	R	SECV
Caffeine	309	0.28	0.14	0.034	0.94	0.043
Theobromine	313	0.97	0.2	0.07	0.88	0.084
Dry matter	772	94.07	0.91	0.14	0.98	0.17
Fat	431	55.58	2.67	0.81	0.91	0.93
B2	192	0.12	0.11	0.04	0.87	0.05
B5	188	0.04	0.04	0.01	0.94	0.02
Epicatechin	187	0.43	0.42	0.08	0.96	0.18
C1	158	0.19	0.19	0.08	0.81	0.11

n; number of replicate measurements, Mean; arithmetic mean of all n measurements, SD; standard deviation, SE; standard error, R; coefficient of determination, SECV; standard error of cross validation.

Only those fermented samples that fulfilled the criteria of adequate fermentation; i.e., presence of the corresponding structure, color and scent of fermented beans when cut in a cutter board, absence of mold and other defects, were included in the investigations.

### Sensory Evaluations

Cocoa liquor was prepared by roasting fermented beans (minimum 300 g per sample) at 120°C for 25 minutes, hand shelling, coarse grinding in a Limprimita cocoa breaker (Capco, Ipswich, UK), and making into a smooth paste for 30 minutes in a heated (48°C) rotating mortar (Pascall Engineering). The paste was then processed to liquor of 20 µm particle size in a 3-roll refiner (Exact). The finished liquor was maintained until use in a preserving jar with an airtight twist-off top. A sample consisting of 100–150 g liquor (depending on the sample size available) was stored throughout the 2-month duration of the replicated tastings. For a single tasting, 30 ml (corresponding to 15 g) of liquor were separated into a glass to avoid contact with odor from any solvents, adhesives and polymers. The sensory tests were carried out at 48°C sample temperature. A panel of ten experienced cocoa tasters that were trained according to the AFNOR (http://www.afnor.org/) standards NF ISO 3972 and NF EN ISO 8586-2 evaluated all samples in three replicates. Scores ranging from 0 (insufficient or negative) to 5 (perfect or positive) were applied as three-replicate averages to 14 attributes. Assessments by individual tasters that diverged greatly from those of the other panel members were excluded. Two-factor (factors taster and sensory attribute, three replicates) analyses of variance and subsequent Newman-Keuls comparisons of means were done to identify individual tasters whose assessments diverged significantly (P<0.05) from those of the other panel members. Grubb’s test [18] was used to detect and exclude extreme values (P<0.05) among the three replicates.

Scores were applied to the sensory attributes acidity, bitterness, astringency, sweetness, taste of cocoa, fresh and dry fruit (similarity with fresh citrus fruit and dry apricot, plum), unroasted and dry seed (similarity with almond or hazelnut), floral aromas (as is known of the Arriba Nacional cocoa of Ecuador), spiciness, and the aroma of fresh wood or sawdust rich in tannins. Attributes that contributed positive aspects were sweetness, acidity, bitterness, cocoa taste, fresh and dry fruit, seed and floral aromas, and spiciness. Attributes that contributed undesired sensations at increased intensities included acidity, bitterness, astringency and wood aroma. A score was also applied to the overall aroma intensity, AI (0; no aroma, 5; very intense AI). An overall “global” score representing the synopsis of all positive and negative sensations was also assigned to the samples. The tasters judged the sensory impression of a sample with respect to what they expected to make up high-quality cocoa. The global score represents a holistic, hedonic approach. It is an assessment based on all positive and negative sensations including acidity and bitterness, good and off flavor resulting from the mixed sensory effects of all attributes. It reflects a synthesis above all individual descriptors. Our panel members are expert cocoa judges. They are trained continuously on fine and also bulk cocoa reference samples thereby making sure that the experts evaluate the global score appropriately.

### Assessment of the Potential Yield

During the harvests, all fruits on the tree were counted that had passed the 12-week development stage, after which abortion of young fruits due to physiological imbalances known as cherelle wilt no longer occurs (p. 87 in [19]). The average number of beans per fruit and the average weight of dry cocoa beans (<8% water content) were used as a reference to assess the tree’s total yield potential (dry cocoa). Losses due to mechanical fruit damage and fruit diseases were not considered. The fact that the harvests were made at different times throughout the harvest period was not considered either. Cacao trees bloom and develop fruits year-round, however, there are two pronounced flushes, and the time of maturation is 140–170 days [8].

### Statistical Analyses

Normal-distribution of the traits scored was tested in the univariate procedure in SAS [20] and Pearson’s correlations were run in the corr procedure. The significance of factors for the performance of individual traits was determined in analyses of variance of the models Trait = Factor+Replication (Tree was the factor and harvest 1 and 2 were the replication term) or Trait = Factor 1+ Factor 2+ (Factor 1×Factor 2)+Replication (factors were individual tree, genotype, harvest, or farm, replications were either trees belonging to the individual genotypes or trees within each farm. For these analyses, the SAS glm procedure with the SS3 option in the model statement was used. Multiple comparisons of least-squares means were carried out with the multiple t-test (tdiff) option in the lsmeans statement, to account for unequal replication numbers.

## Supporting Information

Table S1 Study trees and their cocoa; grouping by genetic background, potential yield, contents of taste determining compounds in fermented and non-fermented beans estimated by NIRS, and results of sensory evaluations throughout two harvests.
(XLS)
